# Child Abuse as an Example of Coexistence of Emotional and Physical Trauma Among Children: An Academic Overview With Altmetric Perspective

**DOI:** 10.7759/cureus.23347

**Published:** 2022-03-21

**Authors:** Fatih Akova, Zehra Koyuncu, Elif Erol, Burak Dogangun, Nuket Guler Baysoy, Mehmet Dokur, Alper Ozkilic, Mehmet Karadag

**Affiliations:** 1 Department of Pediatric Surgery, Biruni University, Istanbul, TUR; 2 Department of Child and Adolescent Psychiatry, Istanbul University-C, Cerrahpasa Medical Faculty, Istanbul, TUR; 3 Department of Psychology, Rumeli University, Istanbul, TUR; 4 Department of Public Health, Biruni University, Istanbul, TUR; 5 Department of Emergency Medicine, Biruni University, Istanbul, TUR; 6 Department of Pediatrics, Biruni University, Istanbul, TUR; 7 Biostatistics and Medical İnformation, School of Medicine, University of Hatay Mustafa Kemal, Hatay, TUR

**Keywords:** altmetric analysis, violence, trauma, social attentions, child abuse

## Abstract

Introduction: Child abuse combines emotional, physical, sexual, and neglect aspects of violence, thus diversifying the trauma for a child. Publications about child abuse had been discussed in academia for long years and evaluated by bibliometric analysis, frequently. This study aims to evaluate the most discussed/disseminated scientific publications about child abuse in electronic media such as social media accounts, blogs, podcasts, and media news sites using a new analysis method called altmetric analysis.

Methods: The data were obtained from the Altmetric Explorer database using the phrase “child abuse,” in 2021. After being ranked by altmetric attention score (AAS), descriptive statistics for all publications as well as detailed analyses for the first-100 publications were performed. Variables evaluated were AAS, dimensions-badge value, distribution of web sources, demographic/geographic-breakdown type distributions, main subject categories, and mesh terms. Kruskal Wallis test was used for AAS and dimensions-badge value comparisons while Spearman correlations and regression analysis were also performed. Analyses were performed by SPSS 23.0 (IBM SPSS, Inc., Chicago, IL, USA) and p<0.05 was considered statistically significant.

Results: Publications about child abuse were shared extensively on social media, mostly on Twitter. In terms of the main subject, sexual abuse was the trending topic, followed by physical abuse and maltreatment. Psychology, studies in human society, health sciences, and law/legal issues were the four main science categories about the subject. The United States was the major disseminator of publications while Child Abuse and Neglect was the most productive journal. There was a weak but significant (p<0.05) positive correlation between AAS and dimensions-badge values.

Conclusion: Child abuse is a multidimensional subject in social media. As the number of publications increases, the possibility of articles to be shared on different social media platforms also increases. The majority of the top-100 publications are the ones emphasizing the importance of child abuse in terms of the prevalence, individual/social burden, and negative consequences.

## Introduction

Child maltreatment or child abuse is a worldwide problem that may lead to serious and lifelong negative effects. All forms of abuse and neglect against minors under the age of 18 constitute child maltreatment. To be more precise, any form of physical and/or biological abuse, sexual exploitation, negligence, apathy that has potential ill-effect on the child`s health, life, and development and applied by who is in a relationship of trust, dependency, or power with a child is considered in the context of maltreatment. Not taking action in a situation that may harm a child is also considered maltreatment. Studies show that about three out of four children between 2 and 4 years of age have been subjected to psychological violence and/or physical punishment regularly by their parents or caretakers [1].

Child neglect and abuse can be classified according to their fatal and non-fatal consequences [2] or depending on whether the treatment of the child is physical, sexual, or emotional [3]. The view is that psychological abuse, also called emotional abuse, is the most common type of abuse and is gradually increasing. On the other hand, attitudes and behaviors that may cause emotional harm to the child in the relationship with the caregiver are considered psychological abuse. This form of abuse is an area where awareness is insufficient compared to its frequency, less known than sexual and physical abuse, and information on its causes, consequences, prevention, and treatment is limited [4]. All kinds of sexual contact, exhibitionism, or sexual harassment against a person who cannot give consent or where there is no real consent, is called sexual violence. The use of the term abuse in this context refers to the disproportionality and severe unjustness of an act against a child in terms of power relations, lack of real consent, vulnerability, and inequality of the child [5,6]. Since the definition of sexual abuse differs according to the culture and laws, its prevalence also varies according to the accepted definition [1]. In a study investigating lifetime sexual abuse exposure of adolescents based on self-report, it was reported that 11.2% of women and 1.9% of men were exposed to sexual abuse by an adult. It has been found that the frequency of sexual abuse increases with age in adolescence [7]. Physical abuse is defined as an act that results in physical harm by a person who is responsible for the child or who is in a relationship of power or trust. Not doing anything for a potentially harmful situation is also included in the scope of physical abuse [8]. It is known that the frequency of physical abuse against children varies in a wide range according to the culture in which it is researched. In a meta-analysis in which data from 168 studies were analyzed, the combined prevalence of physical abuse towards children was reported as 17.7% [9]. Apart from these, any action that results in harm to the child, such as child labor, prostitution, and that takes place within the framework of power disproportion is considered child abuse [8]. Neglect is the failure of the caregiver to use the necessary and accessible opportunities for the child's development. Neglect can occur in areas such as health, education, emotional development, nutrition, and shelter; it may also adversely affect the child's health, physical, mental, spiritual, moral, or social development. In other words, neglect is the failure to supervise children properly and to protect them from harm as much as possible [8]. The prevalence of child neglect was reported as 16.3% for physical neglect and 18.4% for emotional neglect; therefore it stands out as a common and important problem [10].

Child abuse had been discussed in academia for long years and evaluated by citation-based metrics, namely bibliometric studies, quite a lot [11]. However, in this scientific study with the theme of child abuse based on social attention, we used a relatively new alternative analysis method called “article-level metrics” or “altmetrics” [12] and we try to exhibit the complementary social media aspect of the topic. Contrary to citation-based metrics (such as journal impact factor and h-index) which reflect the citing dynamics of articles or books, this new method additionally detects, lists, and evaluates articles most discussed in electronic literature, social media accounts, blogs, podcasts, and news media [13,14]. The score called “Altmetric Attention Score” (AAS) in altmetrics means a publication rises as more people mention it. It simply shows where citations originate from; namely, they originate from public policy documents, mainstream media, Wikipedia, blogs, citations (including Web of Science), social media (Twitter, LinkedIn, Google+, Sina Weibo, and Pinterest) or Multimedia and other online platforms (YouTube, Reddit, etc.) [15]. Eventually, this article gives insight into what is popular in social media about child abuse and explains the dissemination dynamics of the related articles.

## Materials and methods

Data collection

In this scientific research, AAS data in Altmetric Explorer since 2010 were analyzed. The data were obtained from the Altmetric Explorer (available from https://www.altmetric.com) database using the phrase “child abuse” (Date of access: January 5, 2021). Then, all publications on child abuse were ordered from highest to lowest AAS, to reflect the real rating of articles. Each of the writers (pediatric surgeon, pediatrician, child, and adult psychiatrists, public health and emergency medicine professionals) peer-reviewed all publications and select the topic-related articles with consensus. Analysis of the main subject categories and mesh terms enabled writers to avoid keyword-related misses. Besides, among these, the main ideas and Mesh Terms of the top 100 articles were analyzed.

Statistical analysis 

Descriptive statistics (mean ± standard deviation for numerical variables, min-max, and number and percentile values for categorical variables) of all identified publications were performed in this study. Additionally, the top-100 publications with the highest AAS were evaluated with further analyses. Associations between altmetric parameters, namely AAS (an automatically calculated weighted count of all of the attention a research output has received in social media) and dimensions badge value (calculated by interactive visualizations that showcase the citation data origins for individual publications) were identified. The monthly comparisons of AAS and dimensions badge values were made with the Kruskal Wallis test and Dunn test used for post hoc tests. Spearman correlation coefficients were calculated to detect the linear relationship between numerical variables. The correlation coefficients were interpreted as follows: less than 0.4, weak; 0.4-0.6, moderate; 0.6-0.8, strong and 0.81-1.00 high strong relationship [16]. Beta coefficients were estimated by univariate linear regression analysis.

Ethical statement

This study was approved by the University Ethical Committee with the approval number 2021/57-10. All authors declare that the research was conducted following the World Medical Association Declaration of Helsinki's “Ethical Principles for Medical Research Involving Human Subjects.”

## Results

General analysis results for all publications

We discovered 9,534 publications using the phrase “child abuse” in our Altmetric Explorer search. Among all, 9,411 papers were analyzed after excluding the unrelated topics. The average Altmetric score for publications was 7.32± 26.32. (851-0) whereas the average dimensions badge score of the publications was 30.04± 79.53 (0-2431). Authors, titles, AAS scores, dimensions-badge values, and the rankings of the top-100 publications can be seen in Table 1.

**Table 1:** The general characteristics of the top 100 articles on child abuse

Rank	Title	Journal/Collection Title	Altmetric Score	Dimensions
1	The economic burden of child maltreatment in the United States and implications for prevention	Child Abuse & Neglect	851	588
2	Association of Friday School Report Card Release With Saturday Incidence Rates of Agency-Verified Physical Child Abuse	JAMA Pediatrics	592	5
3	Under-ascertainment from healthcare settings of child abuse events among children of soldiers by the U.S. Army Family Advocacy Program	Child Abuse & Neglect	558	5
4	The Lifetime Prevalence of Child Sexual Abuse and Sexual Assault Assessed in†Late Adolescence	Journal of Adolescent Health	531	250
5	The economic burden of child sexual abuse in the United States	Child Abuse & Neglect	530	37
6	An Evidence-Based Education Program for Adults about Child Sexual Abuse (ìPrevent It!î) That Significantly Improves Attitudes, Knowledge, and Behavior	Frontiers in Psychology	467	5
7	Spanking and young childrenís socioemotional development in low- and middle-income countries	Child Abuse & Neglect	461	16
8	The current prevalence of child sexual abuse worldwide: a systematic review and meta-analysis	International Journal of Public Health	444	347
9	Spanking and subsequent behavioral problems in toddlers: A propensity score-matched, prospective study in Japan	Child Abuse & Neglect	434	18
10	Spanking and adult mental health impairment: The case for the designation of spanking as an adverse childhood experience	Child Abuse & Neglect	408	77
11	Effect of Gonadotropin-Releasing Hormone Antagonist on Risk of Committing Child Sexual Abuse in Men With Pedophilic Disorder	JAMA Psychiatry	371	8
12	Beyond the Physical Incident Model: How Children Living with Domestic Violence are Harmed By and Resist Regimes of Coercive Control	Child Abuse Review	358	89
13	Michael Jackson: as an expert in child sexual abuse here's what I thought when I watched Leaving Neverland	The Conversation	347	0
14	When Coercive Control Continues to Harm Children: Post?Separation Fathering, Stalking and Domestic Violence	Child Abuse Review	343	2
15	Asian grooming gangs: how ethnicity made authorities wary of investigating child sexual abuse	The Conversation	337	0
16	Intergenerational transmission of child abuse and neglect: Real or detection bias?	Science	333	155
17	Child sexual abuse	Forensic Science International	329	37
18	Cycle of child sexual abuse: Links between being a victim and becoming a perpetrator	British Journal of Psychiatry	314	134
19	Sexually transmitted infections in children as a marker of child sexual abuse and direction of future research	Current opinion in infectious diseases	296	9
20	Child sexual abuse in religiously affiliated and secular institutions: a retrospective descriptive analysis of data provided by victims in a government-sponsored reappraisal program in Germany	BMC Public Health	269	41
21	Association of a History of Child Abuse With Impaired Myelination in the Anterior Cingulate Cortex: Convergent Epigenetic, Transcriptional, and Morphological Evidence	American Journal of Psychiatry	264	51
22	The Long-Term Health Consequences of Child Physical Abuse, Emotional Abuse, and Neglect: A Systematic Review and Meta-Analysis	PLOS Medicine	231	1341
23	Do adult mental health services identify child abuse and neglect? A systematic review	International Journal of Mental Health Nursing	229	33
24	Verbal abuse during pregnancy increases frequency of newborn hearing screening referral: The Japan Environment and Childrenís Study	Child Abuse & Neglect	213	1
25	Child abuse and mental disorders in Canada	Canadian Medical Association Journal	210	209
26	Emergency Department Admissions for Child Sexual Abuse in the United States From 2010 to 2016	JAMA Pediatrics	210	1
27	Child sexual abuse prevention: What offenders tell us	Child Abuse & Neglect	202	258
28	Community Poverty and Child Abuse Fatalities in the United States	Pediatrics	199	30
29	Risk factors for unidirectional and bidirectional intimate partner violence among young adults	Child Abuse & Neglect	194	143
30	What parents need to know about the signs of child sexual abuse	The Conversation	186	0
31	Child sex abuse doesn't create paedophiles	The Conversation	180	0
32	Emotional and Sexual Correlates of Child Sexual Abuse as a Function of Self-Definition Status	Child Maltreatment	178	12
33	Trends in U.S. Emergency Department Visits Related to Suspected or Confirmed Child Abuse and Neglect Among Children and Adolescents Aged <18 Years Before and During the COVID-19 Pandemic ó United States, January 2019ñSeptember 2020	MMWR: Morbidity & Mortality Weekly Report	175	0
34	Ameliorating the biological impacts of childhood adversity: A review of intervention programs	Child Abuse & Neglect	171	26
35	Stress and parenting during the global COVID-19 pandemic	Child Abuse & Neglect	168	24
36	Legal Proof of Child Sexual Abuse in the Absence of Physical Evidence	Pediatrics	168	0
37	Teenage depression: If a parent doesn't get treatment for a child, is that abuse?	The Conversation	162	0
38	Contextual Risk, Individualised Responses: An Assessment of Safeguarding Responses to Nine Cases of Peer-on-Peer Abuse	Child Abuse Review	160	10
39	Annual Research Review: Enduring neurobiological effects of childhood abuse and neglect	Journal of Child Psychology & Psychiatry	156	442
40	Study: Pandemic-induced stress could be increasing the risk of child abuse	The Conversation	154	0
41	Preventing child sexual abuse : evidence, policy and practice		154	105
42	Long-term Cognitive, Psychological, and Health Outcomes Associated With Child Abuse and Neglect	Pediatrics	154	0
43	The causes of paedophilia and child sexual abuse are more complex than the public believes	The Conversation	154	0
44	What's in a name? Online child abuse material is not 'pornography'	The Conversation	154	0
45	Neuroimaging of child abuse: a critical review	Frontiers in Human Neuroscience	153	387
46	Recovered memories of abuse in women with documented child sexual victimization histories	Journal of Traumatic Stress	152	23
47	School-based education programmes for the prevention of child sexual abuse	Cochrane database of systematic reviews	151	89
48	Teacher Sexual Misconduct: Grooming Patterns and Female Offenders	Journal of Child Sexual Abuse	149	50
49	Association of Child Abuse Exposure With Suicidal Ideation, Suicide Plans, and Suicide Attempts in Military Personnel and the General Population in Canada	JAMA Psychiatry	148	72
50	Childhood emotional abuse, negative emotion-driven impulsivity, and alcohol use in young adulthood	Child Abuse & Neglect	147	27
51	What Influences Believing Child Sexual Abuse Disclosures? The Roles of Depicted Memory Persistence, Participant Gender, Trauma History, and Sexism	Psychology of Women Quarterly	147	23
52	No Surprise: The Rate of Fatal Child Abuse and Neglect Fatalities Is Related to Poverty	Pediatrics	146	3
53	Body mass index and anxiety/depression as mediators of the effects of child sexual and physical abuse on physical health disorders in women	Child Abuse & Neglect	141	28
54	Exposing School Employee Sexual Abuse and Misconduct: Shedding Light on a Sensitive Issue	Journal of Child Sexual Abuse	138	5
55	Pope's child abuse tribunal won't get the Catholic Church out of trouble	The Conversation	138	0
56	Review: Spotlight's revealing story of child abuse in my home town ñ and maybe yours	The Conversation	137	0
57	Child sexual abuse: hearing the cry for help is not always a simple task	The Conversation	136	0
58	A Descriptive Analysis of Public School Educators Arrested for Sex Offenses	Journal of Child Sexual Abuse	136	30
59	Disparities in Child Abuse Victimization in Lesbian, Bisexual, and Heterosexual Women in the Nurses' Health Study II	Journal of Women's Health (15409996)	135	104
60	Disclosure of Child Sexual Abuse Among Adult Male Survivors	Clinical Social Work Journal	135	81
61	Sexual Abuse and Assault in a Large National Sample of Children and Adolescents	Child Maltreatment	135	6
62	Factors related to the reporting of childhood rape	Child Abuse & Neglect	133	92
63	We knew George Pell was guilty of child sex abuse. Why couldn't we say it until now?	The Conversation	133	0
64	Is Exposure to Secondhand Smoke Child Abuse? Yes	Annals of Family Medicine	133	6
65	Child sexual abuse and subsequent psychopathology: results from the National Comorbidity Survey	American Journal of Public Health	132	843
66	Silence of male child sexual abuse in India: Qualitative analysis of barriers for seeking psychiatric help in a multidisciplinary unit in a general hospital	Indian Journal of Psychiatry	130	3
67	African American perspectives on racial disparities in child removals	Child Abuse & Neglect	129	2
68	Unpacking the impact of adverse childhood experiences on adult mental health	Child Abuse & Neglect	129	136
69	A profile of suspected child abuse as a subgroup of major trauma patients	Emergency Medicine Journal	126	25
70	A Standard of Care for the Prevention of Sexual Misconduct by School Employees	Journal of Child Sexual Abuse	126	2
71	Educator Sexual Abuse: Two Case Reports	Journal of Child Sexual Abuse	125	9
72	A Global Perspective on Child Sexual Abuse: Meta-Analysis of Prevalence Around the World	Child Maltreatment	121	888
73	In the Best Interests of the Abuser: Coercive Control, Child Custody Proceedings and the ìExpertî Assessments That Guide Judicial Determinations	Laws	120	17
74	Psychopathology in a large cohort of sexually abused children followed up to 43 years	Child Abuse & Neglect	120	273
75	The relation between dimensions of maltreatment, placement instability, and mental health among youth in foster care	Child Abuse & Neglect	117	15
76	The Politics of Child Abuse in America		114	0
77	Who's Watching the Children? Caregiver Features Associated with Physical Child Abuse versus Accidental Injury	Journal of Pediatrics	110	3
78	An epidemiological overview of child sexual abuse	Journal of Family Medicine and Primary Care	110	54
79	Blamed for being abused: an uncomfortable history of child sexual exploitation	The Conversation	109	0
80	Child Abuse, Sexual Assault, Community Violence and High School Graduation	Review of Behavioral Economics	109	3
81	Child protection report lacks crucial national detail on abuse in out-of-home care	The Conversation	108	0
82	Victims of child sex abuse still face significant legal barriers suing churches - here's why	The Conversation	108	0
83	Questioning the use of adverse childhood experiences (ACEs) questionnaires	Child Abuse & Neglect	107	7
84	The impact of exposure to domestic violence on children and young people: A review of the literature	Child Abuse & Neglect	105	753
85	Jelena Dokic's story of abuse shows links between elite sport and child labour	The Conversation	105	0
86	Does Breastfeeding Protect Against Substantiated Child Abuse and Neglect? A 15-Year Cohort Study	Pediatrics	105	119
87	Royal commission recommends sweeping reforms for Catholic Church to end child abuse	The Conversation	105	0
88	Child Custody Outcomes in Cases Involving Parental Alienation and Abuse Allegations	SSRN Electronic Journal	104	7
89	Is bullying worse than child abuse when it comes to mental health?	The Conversation	104	0
90	Child Abuse Awareness Month During the Coronavirus Disease 2019 Pandemic	JAMA Pediatrics	103	13
91	Moving research beyond the spanking debate	Child Abuse & Neglect	103	10
92	Sentinel Injuries in Infants Evaluated for Child Physical Abuse	Pediatrics	102	145
93	Promising intervention strategies to reduce parentsí use of physical punishment	Child Abuse & Neglect	100	33
94	When it comes to redress for child sexual abuse, all victims should be equal	The Conversation	100	0
95	Child sex abuse survivors are five times more likely to be the victims of sexual assault later in life	The Conversation	98	0
96	New Directions in Child Abuse and Neglect Research	NAP	97	84
97	The impact of child sexual abuse on health: A systematic review of reviews	Clinical Psychology Review	97	491
98	The national apology to victims of institutional child sexual abuse matters. Here's why	The Conversation	97	0
99	Clinical Considerations Related to the Behavioral Manifestations of Child Maltreatment	Pediatrics	97	23
100	Major trauma from suspected child abuse: a profile of the patient pathway	Emergency Medicine Journal	94	11

Mentions, journals, and outputs

**Figure 1:**
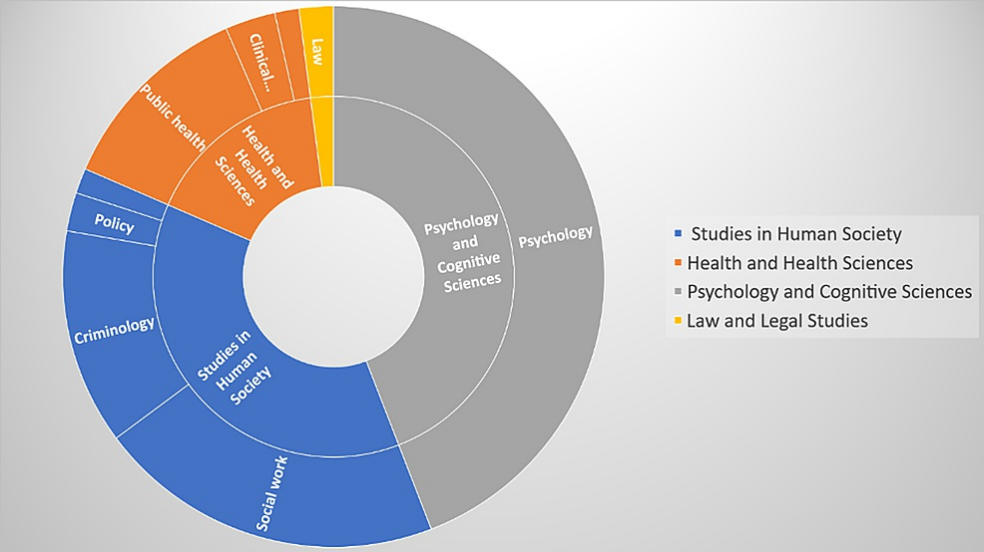
Web of Science categories of child abuse publications disseminated in social media

When document types were analyzed, the majority of the publications disseminated in social media about child abuse were articles (n=8,365), followed by chapters (n=645), books (n=327), news (n=72), and clinical trial data (n=2). When top-100 publications about child abuse were analyzed based on their mentions, the most often mentioned social media platforms were Twitter, Policy, News, and Facebook, respectively. Demographics of mentions were summarized in Table 2. Web of Science categories of child abuse publications disseminated in social media is given in Figure 1. Mainly four categories were detected: psychology and cognitive sciences followed by studies in human society (social work, criminology, and policy), health and health sciences (public health and clinical sciences), and law and legal issues.

**Table 2:** Demographics of mentions

	Mean	SD	Median	Min-Max
Twitter	4.67	19.82	1	0-708
Policy	0.72	1.96	0	0-56
News	0.35	2.77	0	0-103
Facebook	0.18	0.86	0	0-30
Blog	0.07	0.33	0	0-8
Wikipedia	0.07	0.35	0	0-8
Syllabi	0.07	1.16	0	0-70
Google+	0.03	0.86	0	0-78
Peer review	0.02	0.29	0	0-23
Patent	0.01	0.14	0	0-5
Reddit	0.01	0.15	0	0-5
Video	0.01	0.1	0	0-4
Weibo	0	0.02	0	0-2
LinkedIn	0	0.01	0	0-1
Pinterest	0	0.01	0	0-1
F1000	0	0.03	0	0-1
Questions&Answers	0	0.04	0	0-1

**Figure 2:**
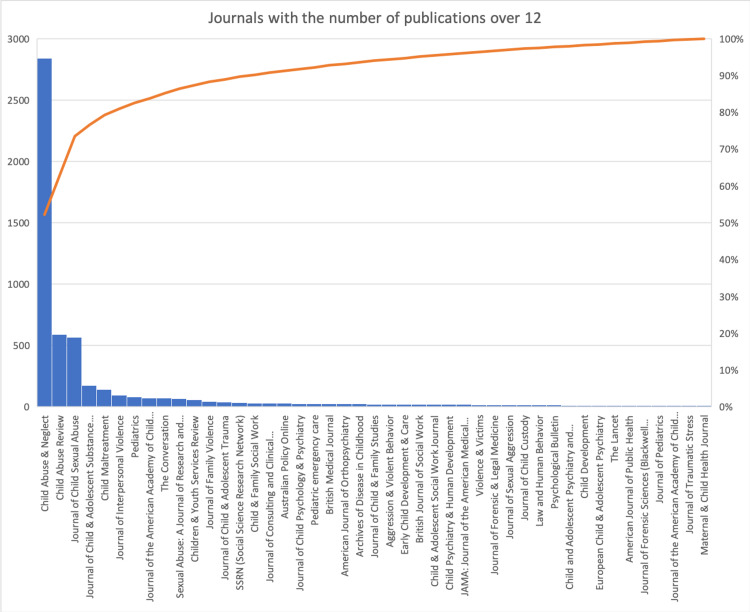
Journals with the number of publications over 12

The top three journals related to the topic of child abuse were Child Abuse & Neglect (n=2,837), Child Abuse Review (n=590), and Journal of Child Sexual Abuse (n=565). Top-20 journals were given in Table 3. The distributions of the number of mentions in each social media platform by journals (for the top five journals) were summarized in Table 4. In each social media platform, 24.6%-39.5% of the disseminations were attributed to the journal named Child Abuse & Neglect. Journals with a number of publications over 12 are summarized in Figure 2. 

**Table 3:** Top-20 journals (articles only)

	number of mention	%
Child Abuse & Neglect	2837	39.7
Child Abuse Review	590	8.2
Journal of Child Sexual Abuse	565	7.9
Journal of Child & Adolescent Substance Abuse	172	2.4
Child Maltreatment	141	2
Journal of Interpersonal Violence	96	1.3
Pediatrics	82	1.1
Journal of the American Academy of Child & Adolescent Psychiatry	73	1
The Conversation	70	1
Sexual Abuse: A Journal of Research and Treatment	66	0.9
Children & Youth Services Review	59	0.8
Journal of Family Violence	45	0.6
Journal of Child & Adolescent Trauma	39	0.5
SSRN (Social Science Research Network)	35	0.5
Child & Family Social Work	30	0.4
Journal of Consulting and Clinical Psychology	30	0.4
Australian Policy Online	29	0.4
Journal of Child Psychology & Psychiatry	27	0.4
Pediatric emergency care	27	0.4
British Medical Journal	26	0.4

**Table 4:** Number of mentions by journals (for the top five journal only)

Mentions & Journals	n	Percent (%)
Twitter Mentions		
Child Abuse & Neglect	15,860	37
The Conversation	4,601	10.7
Child Abuse Review	4,085	9.5
Journal of Child Sexual Abuse	2,430	5.7
Pediatrics	814	1.9
Policy Mentions		
Child Abuse & Neglect	3,107	46.8
Child Abuse Review	452	6.8
Journal of Child Sexual Abuse	345	5.2
Child Maltreatment	227	3.4
Journal of the American Academy of Child & Adolescent Psychiatry	180	2.7
News mentions		
Child Abuse & Neglect	1,239	38.1
The Conversation	298	9.2
Journal of Child Sexual Abuse	151	4.6
Pediatrics	147	4.5
Journal of Child & Adolescent Substance Abuse	107	3.3
Facebook Mentions		
Child Abuse & Neglect	393	24
Child Abuse Review	152	9.3
The Conversation	125	7.6
Pediatrics	108	6.6
Journal of Child Sexual Abuse	59	3.6
Blog mentions		
Child Abuse & Neglect	255	39.4
Child Abuse Review	56	8.7
Pediatrics	15	2.3
Child Maltreatment	13	2
Journal of Child Sexual Abuse	12	1.9
Wikipedia		
Child Abuse & Neglect	225	36.5
Journal of Child Sexual Abuse	57	9.2
Child Abuse Review	35	5.7
Child Maltreatment	17	2.8
Sexual Abuse: A Journal of Research and Treatment	12	1.9

Number of posts by countries

**Figure 3:**
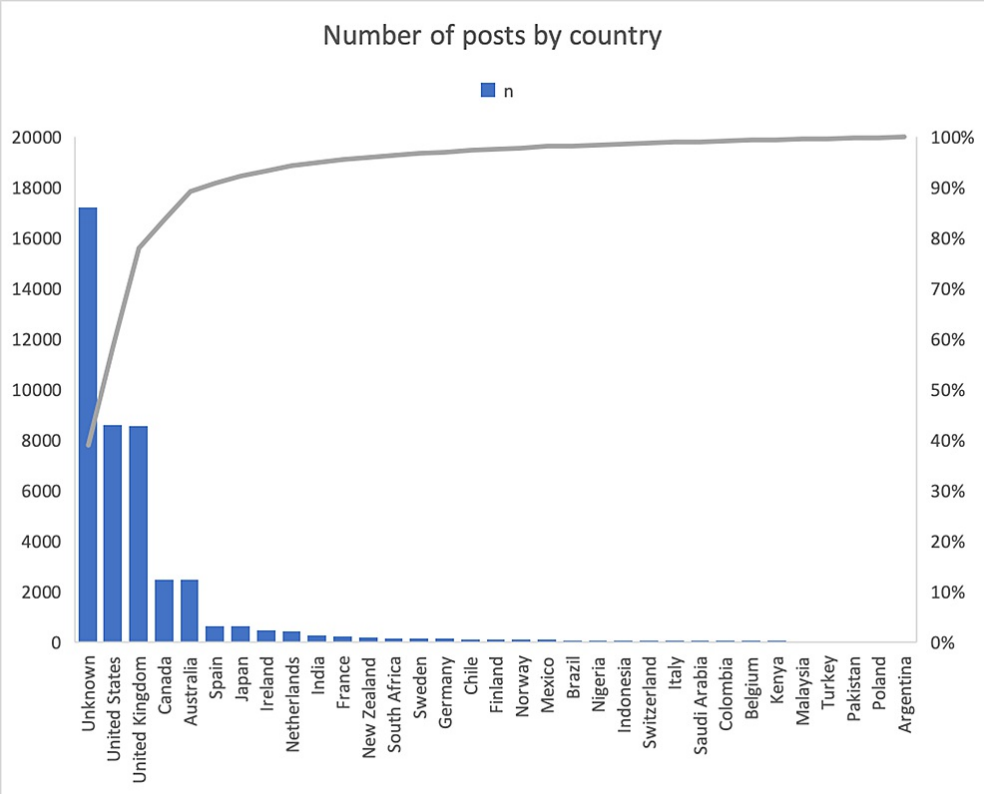
Number of posts by country

The worldwide distribution of posts by country is given in Figure 3. Apart from unknown origins, the top-3 countries producing the utmost dissemination were the United States, the United Kingdom, and Australia.

Analysis of the top 100 publications with the highest AAS

The average altmetric attention score for the top 100 publications with the highest AAS (T100 list) was 202.27± 134.33 (98-851). The publication with the highest AAS with 851 was Fang et al.’s article and it was entitled “The economic burden of child maltreatment in the United States and implications for prevention.” On the other hand, the average dimensions for the T100 list were 30.04± 79.53 (0-2,431). The publication with the highest dimensions-badge score (2,431) was Bernstein et al.’s article and it was entitled “Development and validation of a brief screening version of the Childhood Trauma Questionnaire.” Details can be seen in Table 1. 

**Figure 4:**
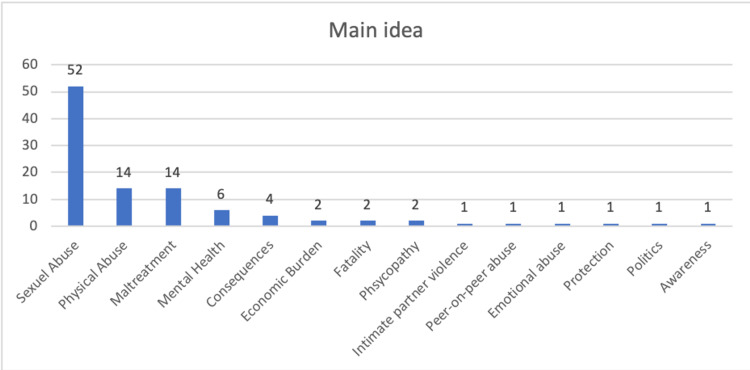
Main ideas of the top 100 articles

**Figure 5:**
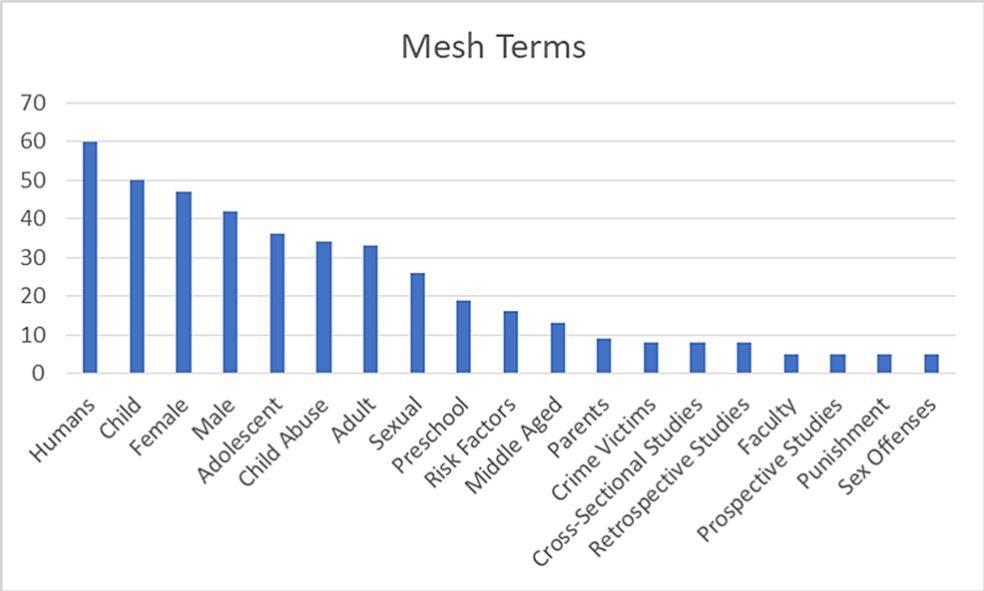
Mesh terms of the top 100 articles

Analysis of the top-100 list main subject category reveals that the majority of articles were related to sexual abuse (n=52), maltreatment (n=14), and physical abuse (n=14) (Figure 4). Mesh terms of the top-100 articles are summarized in Figure 5.

Correlation and regression analysis of the Altmetric score and dimensions-badge values for the T100 list

**Figure 6:**
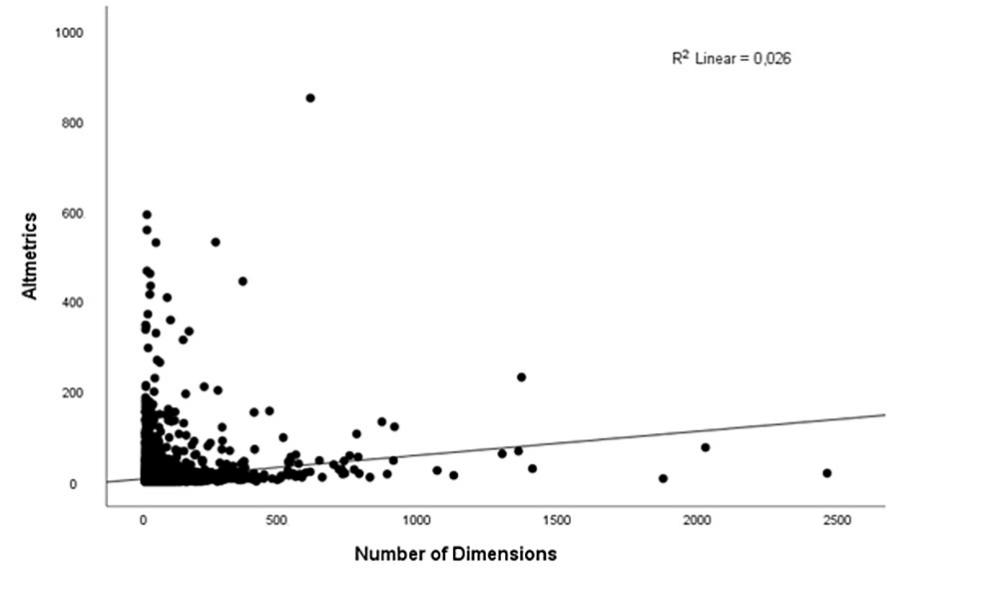
Scatter plot of the relationship between dimensions and altmetric scores

Scatter plots of the relationship between dimensions and altmetric scores were summarized in Figure 6. There was a weak positive correlation between altmetric scores and dimensions-badge values (r=0.161; p=0.001) (Figure 6). Univariate linear regression analysis revealed that 0.26% of the variation in altmetric was explained by dimensions (Table 5). One unit increase in dimensions resulted in a 1.23 unit increase in altmetrics. Regression model to estimate altmetric as follows:

Y Altmetric=1.23*X Dimensions.

**Table 5:** Univariate linear regression analysis result to estimate Altmetric analysis by using citations ^a^Dependent variable

	Unstandardized Coefficient^a^		
	B	Std. Error	P
Constant	2.25	0.27	0.001
Citation	1.23	0.09	0.001

## Discussion

With its different forms of occurrences, child abuse still stands before us as a multifaceted social problem and it is understood that it has been on the agenda in social media for all years since the beginning of the production of altmetric data.

A weak positive correlation between AAS (the weighted count of all of the attention a research output has received) and dimensions-badge (the summary of the web origin of citations about the publication in concern) reminds that the two altmetric parameters are related to each other. This means that the shares of the publications and the prevalence of these shares in different social media account types change simultaneously.

Child abuse concerns a wide range of disciplines in the context of social media. It is understood that there is a lot of sharing in the field of psychology and studies in human society (sociology, politics and administration, social work, and criminology) followed by health sciences (especially public health, clinical sciences, pediatrics, nursing, neurosciences) and law [17,18]. Some very diverse branches also made publications about child abuse such as education, language, communication and culture, economics, philosophy, and religious studies, historical studies, genetics, information and computing sciences, commerce, management, tourism, business, marketing, chemical sciences, journalism and professional writing, biomedical engineering, built environment and design. 

Publications are shared extensively on social media, mostly on Twitter. After Twitter, the most disseminated social media area has been policy documents. This is an indication that the official and judicial dimension of the issue constitutes the agenda. Then news occupies an important place, that is, there are publications with news quality, and they are shared widely. It is noteworthy that Facebook and blog posts for child abuse are relatively few. Therefore, the subject is a hot topic both in the scientific community, in official channels, and in social media news channels.

Although scientific disseminations about child abuse originate worldwide, scientifically productive and developed countries such as the United States, United Kingdom, Australia, Canada, Japan, Spain, France generated most of the dissemination. The fact that the issue is an important topic in the United States and the United Kingdom may increase the potential for such research to be shared by these countries [19]. The large body of research on child abuse in these countries may also reflect the great anxiety that the Western world has devoted to it. Higher awareness of children's rights in these countries may also contribute to this situation [19,20]. However, some reports showed that the trend of child abuse was not a true increase in prevalence but due to the changes in legislation [21,22]. In low and middle-income countries, where child abuse is seen as taboo and sensitive, such publications are less common [23]. In a sample of nine countries, according to a research article, the use of corporal punishment for children was lowest by Swedish parents and highest by Kenyan parents [24]. It can also be considered that a subject such as child abuse remains in the background, as low and middle-income countries give more importance to the studies to meet the basic needs of the society.

We see that the publications in the Child Abuse & Neglect journal occupy the most agenda on all social media platforms. It is seen that the Child Abuse & Neglect journal has published articles in this field for a long time (since 1977) and has a high impact factor (reported as 3.9 for 2019) compared to other journals in the ranking. In addition, a high number of annual total articles since it is published monthly and listing of this journal in many indexes can explain the fact that the articles in the journal reach large audiences and take more space in social media.

When the content of the most-disseminated top-100 publications was evaluated in terms of the main subject, sexual abuse was the trending topic (Figure 4). This is followed by physical abuse and maltreatment. Since the legal consequences of sexual abuse are more pronounced and severe; also, it is more documented due to legal processes, it may become a current issue. It does not seem very possible to explain this only from a medicolegal perspective. Even if the consequences of sexual abuse are not very obvious in the early periods in some cases, more apparent problems may arise during the adolescence period when sexual development accelerates or when sexual activity begins. Finally, the most unacceptable form of child maltreatment may be to approach a child sexually. For example, in some cultures, physical violence is considered more normal (There are some proverbs in Turkish culture, for example, those who do not beat their daughter will beat their knees). Since ancient times, we see that the behaviors of daily life, which we today accept as child abuse, were the natural cultural elements of life both in the world and in the Anatolian lands during and before the Ottoman Empire [25]. However, while there may be families who find it normal to physically punish their children for disciplinary purposes, we do not come across a culture that considers sexual behavior towards a child normal [26]. Nevertheless, looking at the marriage statistics in Turkey, 17,058 girls aged 16-17 were married in 2019, and 13,014 girls were married in a civil marriage in 2020 [27]. While marriages before the age of 16 are not reflected in official figures. Again, according to official statistical figures, 8,271 girls under the age of 17 gave birth in 2020 and 10,045 girls in 2019 [28]. These figures can be considered striking indicators of the intertwining of sexual abuse with culture. Therefore, sexual abuse may be becoming more noticeable.

On the other hand, it has been seen that the issue of emotional abuse, which is known to occur a lot, is rarely talked about. Although it is known that emotional neglect and abuse are very common, there are difficulties in documenting them. It is seen that the effect on the psychological and mental health of the victim has also been less studied. From this point of view, it can be thought that emotional abuse and neglect of the child are neglected also scientifically [4]. It can be accepted plausible that a topic containing little data may become less apparent. 

Again, it has been noticed that “awareness of child abuse,” which is considered as an important step in the prevention of abuse, is relatively less discussed in social media. Child neglect and abuse is a common condition that is likely to be found in many people's life histories, and increased awareness can lead to the recollection of individual traumas and stress. From this point of view, inadequate handling of awareness of abuse may be an avoidance mechanism. When considered from another point of view, talking less about awareness can also be evaluated as an effort to keep away from situations that are perceived as bad and dangerous. Increasing awareness of child neglect and abuse reminds us that this is a very common and frequent reality. However, some of the ways to cope with highly emotionally charged situations are to ignore them or to keep the dangerous situation out and away. The underappreciation of awareness may be serving this ignorance [29]. Another reason for the low awareness of abuse may be that the perpetrators are from the family and close circles. In such cases, families avoid this confrontation for reasons such as not experiencing tension in the family, avoiding gossip in the neighborhood, or the emotional shock of the event. According to the results of the Research on Domestic Violence Against Women in Turkey; 9% of women have been sexually abused before the age of 15. While 29% of the perpetrators are male relatives, 15% are reported as people from close circles such as neighbors or grocers [30].

In the evaluation made based on mesh terms, the publication could be evaluated beyond the mere main idea, with its much different dimensions. Evaluation reveals that the most used mesh term is “humans.” This is followed by 'child'. In addition, child abuse was also examined in terms of gender, and focused more on the female. The developmental stage of the victim (preschool, adolescent, middle-aged, adult) has been important when dealing with child abuse. When child abuse is a legal issue, it is also paired with terms such as punishment, sex offenses, and crime victims.

The majority of the top-100 publications are the ones emphasizing the importance of child abuse in terms of the prevalence, individual and social burden, and negative consequences, and reflecting general information about the subject. In addition, there are publications that attracts attention due to their striking results (Table 1; #2, #3, #11, #13, #15, #58, #66, #87). There are reports about the ways that lead to child abuse or the underlying causes of this event, such as the intergenerational transmission of the trauma or the possibility of yesterday's victim being the perpetrator of today (Table 1; #18, #20). An article argued that perpetrators are mostly men, contradicting our preconceived notions (Table 1; #51). Also, a 15-year prospective follow-up study that examines some factors possibly protective for child abuse (Table 1; #88), as well as a study that addresses a current issue such as child neglect and abuse during the COVID-19 pandemic (Table 1; #36) were popular.

Limitations

Although altmetric analysis enables a quantitative look at social media accounts in terms of scientific article disseminations, this method has some limitations. The infrastructure of Altmetric Explorer enables a general look at the investigated subject in both qualitative and quantitative ways and gives an opportunity to investigate every single article individually, but it is not always possible to evaluate a huge number of publications in such a detailed way, usually, top-50 or top-100 evaluated in a detailed way (as in this study). Also, the main dissemination route for Altmetric Explorer is Twitter, thus different platforms might have been less represented in this study.

## Conclusions

Child abuse is a common type of trauma all over the world. It concerns many different scientific disciplines, from health to law, from psychology to sociology, whose effects can last a lifetime, cause negative consequences and, create a social burden. Scientific publications on child abuse are hot-topics in the scientific community, official media channels, and social media news channels. It is understood that Twitter is especially effective in spreading the issue. Child abuse is discussed in social media with many different aspects, but especially with the dimension of sexual abuse. Efforts to prevent child abuse also particularly attract attention.
